# Selection preferences for animal species used in bone-tool-manufacturing strategies in KwaZulu-Natal, South Africa

**DOI:** 10.1371/journal.pone.0249296

**Published:** 2021-04-01

**Authors:** Justin Bradfield, Andrew C. Kitchener, Michael Buckley

**Affiliations:** 1 Palaeo-Research Institute, University of Johannesburg, Johannesburg, South Africa; 2 Department of Natural Sciences, National Museums Scotland, Edinburgh, United Kingdom; 3 School of Geosciences, University of Edinburgh, Edinburgh, United Kingdom; 4 School of Natural Sciences, Manchester Institute of Biotechnology, The University of Manchester, Manchester, United Kingdom; Griffith University, AUSTRALIA

## Abstract

Animal symbolism is a prominent feature of many human societies globally. In some cases, these symbolic attributes manifest in the technological domain, influencing the decision to use the bones of certain animals and not others for tool manufacture. In southern Africa, animals feature prominently in the cosmogenic narratives of both hunter-gatherer and Bantu-speaking farmer groups. Whenever these two culturally distinct groups came into contact with each other there would be an assimilation of cosmogenic concepts of power and the adoption of certain symbolically important animals. In this paper, we report on which animals were selected to make bone tools during the first millennium AD contact period in KwaZulu-Natal Province, South Africa, and explore the extent to which this selection may have been influenced by the symbolic associations of specific animals. Our results show selective targeting of specific animals for tool manufacture at some sites, with a narrowing of the range of selected species during the first millennium AD contact period. Certain antelope tribes, such as Aepycerotini, Cephalophini and Antilopini, appear to have been deliberately avoided, thus arguing against opportunistic selection. Nor does the range of selected animals appear to show any obvious mechanical considerations, as has been noted in similar studies. We highlight the potential of ZooMS for understanding the dynamics of animal symbolism in the past.

## Introduction

Animals have played and continue to play a prominent role in human societies, and are commonly used as metaphors through which to think about and discuss a wide range of human concepts and societal issues [1]. Although cultural conceptions of animals and peoples’ attitudes towards them are diverse and complex, animal symbolism plays a prominent role in articulating social structure among most human societies [2, 3]. Evidence for this may be seen in any number of case studies. For example, among Nigeria’s Yoruba community animal images and metaphors feature prominently in cosmogenic myths and are used to convey concepts and perpetuate traditions, such as clan identity and sacred leadership [4]. A similar situation is seen among many other African groups where animal symbolism is intimately woven into the social fabric, being used to convey concepts of power, healing and protection [5, 6].

The symbolic role of animals in archaeology is well acknowledged and has given birth to the sub-discipline of social zooarchaeology, which explores how animals were integrated into the social and ideological fabric of human life [7–9]. In some cases, the social or symbolic importance of animals would translate into the technological sphere. Preferential selection of certain animal species for tool manufacture is evident among several cultures. For example, the Thule Inuit would make certain classes of tools out of ivory and others out of antler [10]. These selection biases were dictated, not only by function, but by particular cultural considerations [10]. At the Later Stone Age site of Taforalt in Morocco, bone tool manufacture was embedded within culturally mediated strategies whereby certain animals were preferentially selected to make certain types of tools, while other animals were reserved for other types of tools [11]. Neanderthals’ preferential strategic selection of bison ribs to make lissoirs in layers dominated by reindeer remains [12], could also point to a symbolic role of bison in certain parts of France during the Middle Palaeolithic. Likewise, it has recently been found that among the pre-contact St-Lawrence Iroquoians, animal symbolism augmented the practical and functional considerations of bone-tool manufacture [13].

The advent of bone tools in many societies accompanied increased social complexity and technological innovations by participating in flows of social networks and information [14, 15]. Social zooarchaeology aims to address such questions as the symbolic role of animals and how this affected bone selection and bone-working technology among human societies [16]. With some notable exceptions, social zooarchaeology is still in its infancy in southern Africa [17]. Attempts have been made recently to examine possible animal selection strategies in bone-tool manufacture in the 58–65 ka period at Sibudu Shelter, South Africa [18] and during the early period of hunter-gatherer and farmer contact in the north of South Africa [19]. In the first study, it appears that there was a switch from a focus on perissodactyl bone to artiodactyl bone through time, while the latter study showed that people selected a narrower range of species for tool manufacture than for food, and that certain species may have been specifically selected for tool manufacture. The sample size in both studies was unfortunately too small to make confident interpretations or to rule out definitively other selection considerations.

**Fig 1:**
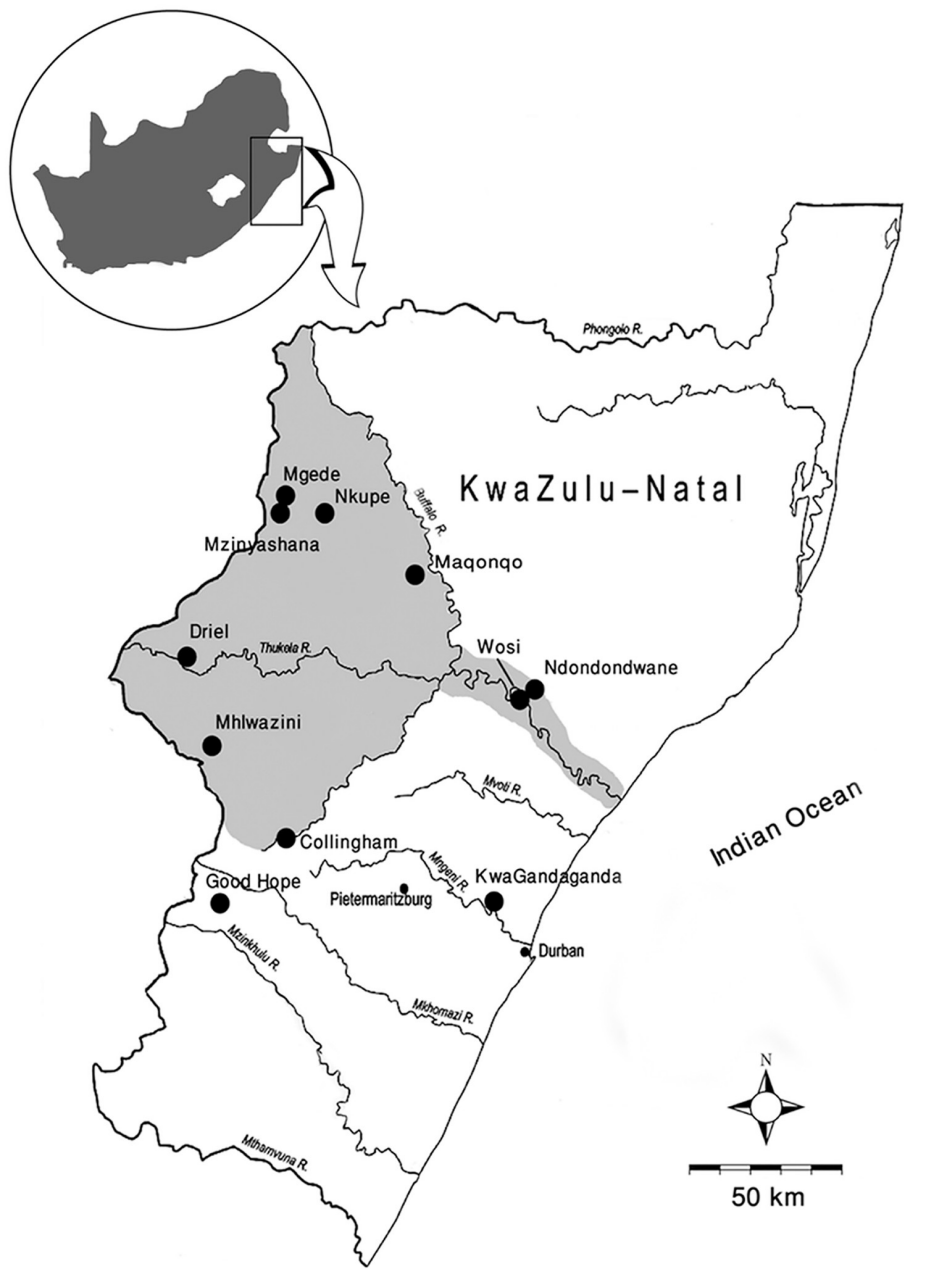
Map showing the sites mentioned in this study. Smaller dots represent the main metropolitan areas in the province. Shaded area roughly represents the Tugela River catchment.

**Fig 2:**
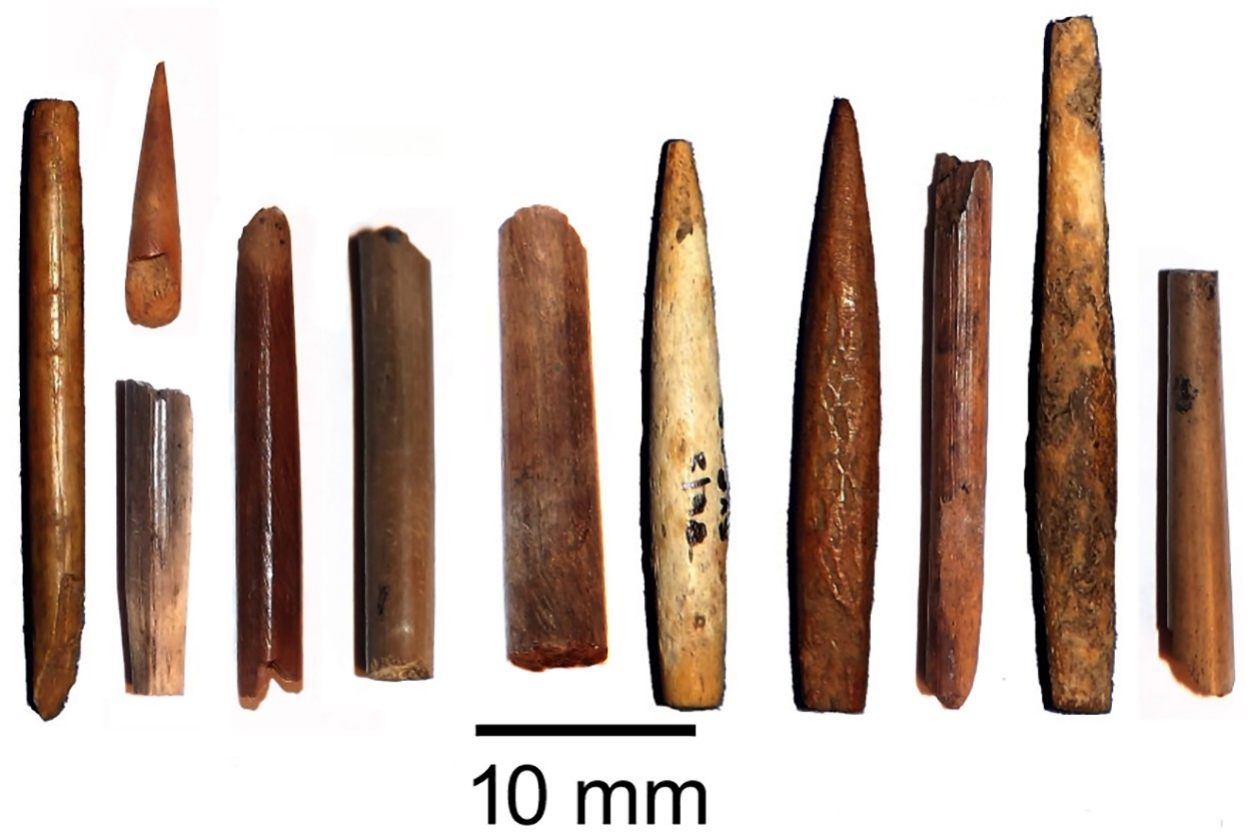
Selection of bone point fragments sampled from Nkupe shelter and KwaGandaganda.

In this paper we offer the first glimpse of the strategic selection of animals for bone-tool manufacture during the first millennium AD contact period in KwaZulu-Natal, South Africa. We look at 84 modified bone tools from 11 Later Stone Age and Early Iron Age sites in the province, mainly from the Tugela River basin (Fig 1). The modified shaft fragments (Fig 2) have been classified as arrowheads by the excavators, but could have served any number of purposes [20]. There has been a lot written about the relations between the autochthonous hunter-gatherers of the region and the first immigrant Bantu-speaking farming communities, particularly the extent to which the cosmology of each group was affected by the other [21–25]. Contact also affected how some animals were seen by each group. We explore the extent to which animal symbolism may have translated into technology among each group, and how this may have shifted or changed during the period of contact.

## Background

### Animal symbolism among the San and Nguni

Animals were important protagonists in the myths and folktales of the San Bushmen [26, 27]. Certain animals, such as the eland and hartebeest, were believed to possess magical powers and contain supernatural potency, which could be harnessed during certain ceremonies [28]. Some shamans were believed to be able to magically manipulate the movement of animals and to influence the outcome of an upcoming hunt [27], a practice also seen among the Yukaghir of Siberia [29]. Some of these ceremonies are depicted in rock art in the form of therianthropic figures [30, 31]. Shamans of the game wore the skins of certain animals, like rhebok or springbuck, and symbolically wounded themselves in order to sympathetically wound the targeted prey [27, 32, 33]. It is plausible that some therianthropic figures represent these shamans rather than visions induced during trance.

Shamans were also thought to be able to control the rain. Kudu are frequently painted in northern South Africa, and are thought to have been important in rain-control ceremonies among both hunter-gatherers and early farmers [34, 35]. Brunton and colleagues [36] describe a wide variety of species believed to be rich in supernatural potency and which were used by farmers in the early second millennium AD in rain control rituals. The species recovered from Ratho Kroonkop, Limpopo Province, include mainly small mammals, birds, reptiles and fishes, but larger animals, including rhinoceros, buffalo, zebra and reedbuck, are also present. The rhinoceros is one such animal that had wide-ranging associations among hunter-gatherers and farmers. For example, rhinoceros depictions are found in hunter-gatherer rock art associated with trance rituals [37], and at farmer sites, where they are associated with rain making [38] and concepts of power and leadership [39].

Among the Nguni and Sotho-Tswana, animal veneration is a defining aspect of rural society, and animals are still used regularly as metaphors of communication [40, 41]. Ancestral spirits are commonly ascribed the behavioural traits of certain wild animals, including elephants, lions, leopards, jackals and baboons [42]. Antelopes are less common, but include the steenbok, duiker, bushbuck, klipspringer and grysbok [42]. Ancestral spirits are believed to commune with Nguni diviners in the form of *ityala* (divinatory animals). Forty-three species are listed as divinatory animals, the most common of which are the lion, leopard and elephant; followed by buffalo, hyaena, bushbuck and springbuck [43, 44]. The bones of some of these animals form an integral component of a diviner’s kit as they are believed to confer the ‘powers’ of the animal to the diviner [42, 45]. Just as among the San, the concept of sympathetic magic was pervasive in Nguni society [43, 46].

Animals were also depicted in figurines and used to transmit information during initiation ceremonies of several Bantu-speaking groups [47]. Some of these zoomorphic figurines and ceramic vessels have been found in the Tugela catchment region and, based on their horns, are thought to resemble reedbuck, waterbuck and roan antelope [48, 49]. Sotho and Nguni ritual functionaries and praise singers also commonly wore headdresses made from animal scalps [50, 51], and it is possible that some therianthropes in the rock art of KwaZulu-Natal depict these figures [52]. The symbolic attributes of aquatic animals are thought to have engendered some of the food-avoidance practices of the Nguni [49, 53].

### Contact and the exchange of ideologies

When two mutually distinct groups, such as hunter-gatherers and farmers, come into contact with each other, there are inevitable interactions and exchanges that take place [52, 54]. There are many historical sources, including oral histories, which document such interactions in South Africa from the sixteenth to nineteenth centuries AD [23, 52, 55–59]. One outcome of these contact situations was the selective adoption by the Nguni of certain cultural beliefs and practices of the San, and *vice versa*, including cosmogenic concepts of power and divinatory animals [24, 60]. The Nguni regarded the San as spiritual mediators, able to intercede with the supernatural world to bring about rain and other boons. The similarity in regalia and paraphernalia between Nguni diviners and San shamans, including animal headdresses, bears testament to the intimate nature of these cultural adoptions [23]. Even the places the San occupied were seen by the Nguni as having supernatural attributes. San rock-art shelters were often used by the Nguni for divinatory rituals [23]. These conceptual borrowings are also evident in linguistic associations, where certain words, including those of animals, like elephant and roan antelope, were adopted from the San by the Nguni [32, 61]. Such was the extent of interaction by the nineteenth century that some San rock artists may have been of mixed Nguni descent [30, 62].

Much has been written about the archaeology of the early period of hunter-gatherer and farmer contact, particularly in Limpopo Province, where evidence suggests the hunter-gatherers were initially employed by the farmers as ritual specialists [63–65]. In KwaZulu-Natal the sustained period of overlap between the hunter-gatherer and farmer occupations suggests symbiotic interactions between the two groups, at least initially [22, 66, 67]. But, if cultural exchanges were initially amicable, they were not one sided [25]. Hunter-gatherer rock art, which intensified during periods of contact, bears testament to farmer influences [68]. The role of eland in hunter-gatherer rock art was substituted by cattle after contact [69]. This appropriation shows that the symbolism attached to animals was fluid and that other animals may have been important at different times in the past. Thus, Thackeray [32] notes that eland, rhebok and roan antelope may have all held similar importance to hunter-gatherers, and would have been painted interchangeably, depending on which species was more prevalent in a particular landscape.

### The Later Stone Age and Early Iron Age of the Tugela River catchment

Many of the KwaZulu-Natal Later Stone Age sites, and most of those included in this study, were excavated by Aaron Mazel as part of an extensive research programme during the 1980s-1990s. Mazel constructed an elaborate social history scheme for the Later Stone Age of the Tugela catchment area. There have been valid criticisms of this scheme [70], but there has never been a comprehensive alternative offered. The following narrative of the archaeology of the region is taken primarily from Mazel’s summative accounts [21, 22], augmented with more recent reports.

There is no recorded settlement of the Tugela catchment area prior to ~10 ka, owing to arid climatic conditions. Thereafter, small-scale, intermittent occupation began in the eastern highlands of the catchment, from whence it expanded after ~7 ka. Between 7–2 ka exploitation of small game, microfauna, underground plant foods, and fishes intensified due to increased sedentism and population growth. Three phases of social re-structuring occurred during this period. From 7–6 ka material culture is uniform at all sites, suggesting a single social alliance network, which had contact with the coast [71, 72]. A period of flux followed, where subsistence started to diversify, with fish being added to the diet. Concomitantly, large bovid exploitation starts to decline. At this time stone scrapers start to get smaller and more worked bone is produced. From just before 4 ka to roughly 2 ka idiosyncratic styles emerge in the material culture. The single social network appears to have split into three, possibly four [73], regional alliance networks, which likely extended beyond the catchment area [74], but which ostensibly avoided the central Tugela River corridor. The Ndaka social region contains Nkupe, Mgede and Mzinyashana shelters, with Driel, Mhlwazini, Collingham and possibly Good Hope shelters, forming part of the Injasuthi social region, and Maqonqo lying in what was originally called the Toleni region, but later thought to be part of the unnamed fourth social region (Fig 1).

Once farming communities began settling in the region in the first millennium AD, hunter-gatherers started moving out of the high-elevation mountainous areas to take up occupation in the previously unoccupied central corridor, close to where the farmer settlements were located. Archaeological and genetic information suggests that initially relations between the two groups were amicable and more equitable than farther north in Limpopo Province [75]. From about 1000 AD onwards evidence for contact lessens. Evidence suggests exchange at this time was uni-directional from farmers to hunter-gatherers. Hunter-gatherer material is absent from the farmer sites, although famers still seem to have frequented rock shelters, which they probably used for ritual purposes [71].

Several sites in the study area contain rock art, the oldest being at Maqonqo ~3.7 ka [73, 76]. Most of the art, however, is thought to date to approximately 2 ka [68]. The most commonly depicted animals in the region are grey rhebok, hartebeest and eland [76, 77]. Before Driel shelter was flooded there was a rock art panel depicting men with spears hunting an elephant [66]. The faunal remains from several sites show a wider variety of species represented than are present in the area today [78]. Some contain species that are known to have been ritually important, for example, pangolins, aardvarks, primates, honey badgers, wild dogs and other carnivores (Table 1; [79]). The latter are more prevalent in the upper layers at sites like Nkupe [80]). In most cases, small animals dominate the remains and, at least at Good Hope shelter, it appears that larger bovids were butchered away from site and only the meat-bearing limbs brought back [81]. The remains of domestic animals are found in the contact-period levels at some sites, which indicate barter with neighbouring farmers, or intermittent stock minding [78, 82]. Another characteristic of the post-2-ka layers at sites such as Mgede, Nkupe and Driel, is that the bone points become faceted. Similar faceted bone points were found at a contact-period farmer site in Limpopo [83]. These were originally thought to have been a cache of unfinished arrowheads, but recent use-trace analysis has shown that they were in fact hafted into reed shafts, so were most likely considered complete by their makers [84].

**Table 1:** Species identified in the faunal analyses at the eleven sites from KwaZulu-Natal included in this study. The numbers represent MNI counts, except where only NISP counts were available, in which case and ’X’ marks species presence. Full NISP data (where available) from the sites can be found in S1 Table. In some cases, the taxonomic names have been updated from what appears in the original fauna reports to accommodate most recent scientific parlance.

	Tribe	Colling.	Driel	GH	Kwa.	Maqon.	Mgede	Mhlwaz.	Mzinya.	Ndond.	Nkupe	Wosi
*Homo sapiens* (**human**)			1			1	1		1		2	
*Papio ursinus* (**chacma baboon**)		7		12		13	1	11	1		18	5
*Chlorocebus aethiops* (**vervet monkey**)					X							4
*Lupulella mesomelas* (**black-backed jackal**)		4	1			15	4	6	7		10	2
*Lycaon pictus* (**wild dog**)						2			2		2	
*Vulpes chama* (**cape fox**)			1			1						
*Canis familiaris* (**dog**)					X					X		8
*Crocuta crocuta* (**spotted hyaena**)									1			
*Parahyaena brunnea* (**brown hyaena**)		1				2						
*Caracal caracal* (**caracal**)		1	1			3	2	1	4		12	2
*Felis lybica* (**wildcat**)		3	1		X	1	4		3		14	2
*Leptailurus serval* (**serval)**												2
*Genetta genetta* (**genet**)						2					7	1
*Genetta tigrina* (**Cape genet**)					X							
*Panthera leo* (**lion**)						3					2	
*Panthera pardus* (**leopard**)		3			X	1	1				2	
*Equus quagga* (**zebra**)				1	X	23	2		5		1	1
*Procavia capensis* (**hyrax**)		29	6	11	X	36	7	11			53	
*Proteles cristatus* (**aardwolf**)			2			3			2			
*Phacochoerus* sp. (**warthog**)			4	1	X	39		1	10		4	4
*Potamochoerus larvatus* (**bushpig**)		2			X	4	5		14		11	4
*Orycteropus afer* (**aardvark**)		1	2			14	2		7		5	3
*Smutsia temminckii* (**pangolin**)					X	4			1			
*Mellivora capensis* (**honey badger**)						1	1				1	
*Giraffa giraffa* (**giraffe**)						1						
*Loxodonta africana* (**African elephant**)					X							X
*Hippopotamus amphibius* (**hippo**)			1		X					X		10
Rhinocerotidae (**white & black rhinoceros**)									1			
*Ovis/Capra* (**sheep and goats**)	Caprini				X	8		1	4	X	1	402
*Aepyceros melampus* (**impala**)	Aepycerotini				X	13		1	8			1
*Alcelaphus caama* (**hartebeest**)	Alcelaphini	3		5		9			2			
*Connochaetes gnou* (**black wildebeest**)	Alcelaphini	1	5				4				4	
*Connochaetes taurinus* (**blue wildebeest**)	Alcelaphini					14			2			1
*Damaliscus pygargus* (**blesbok/bontebok**)	Alcelaphini	1	2	2		2					4	
*Antidorcas marsupialis* (**springbuck**)	Antilopini		1					1	4			
*Neotragus moschatus* (**suni**)	Antilopinae								3			
*Oreotragus oreotragus* (**klipspringer**)	Oreotragini	2		6		17	5	18	9		17	
*Ourebia ourebi* (**oribi**)	Antilopini		6	3		11	4	1	11		22	
*Raphicerus campestris* (**steenbok**)	Antilopinae	3	1		X	29		10	18		11	
*Raphicerus melanotis* (**grysbok**)	Antilopini			3			3					
*Cephalophus natalensis* (**red duiker**)	Cephalophini	1			X			2	6			
*Philantomba monticola* (**blue duiker**)	Cephalophini				X	12		1				11
*Sylvicapra grimmia* (**common duiker**)	Cephalophini	2			X	35		4	8	X		24
*Hippotragus* sp. (**roan and/or sable**)	Hippotragini					3	1		1		1	
*Pelea capreolus* (**grey rhebuck**)	Reduncini	8		3		7	5	16	8	X	21	
*Kobus ellipsiprymnus* (**waterbuck**)	Reduncini								1			
*Redunca arundinum* (**reedbuck**)	Reduncini	2	5			7			10	X		
*Redunca fulvorufula* (**mountain reedbuck**)	Reduncini	2		5		25	3	2	10		14	3
*Taurotragus oryx* (**eland**)	Tragelaphinini	8	2	2		13		3	4			
*Tragelaphus angasii* (**nyala**)	Tragelaphinini									X		
*Tragelaphus sylvaticus* (**bushbuck**)	Tragelaphinini					5	1		2			1
*Tragelaphus strepsiceros* (**kudu**)	Tragelaphinini					4			3			
*Bos taurus* (**cattle**)	Bovini			2	X				5	X		38
*Syncerus caffer* (**buffalo**)	Bovini				X	3			1		3	
*BOV I*		2	7	13	33	12	15	11	5	0	54	10
*BOV II*		3	6	11	37	30	16	12	10	0	63	6
*BOV III*		2	14	7	8	16	7	4	7	0	16	5
*BOV IV*		0	3	5	0	1	3	0	4	0	8	0
*Aonyx capensis* (**clawless otter**)		2									4	2
*Atilax paludinosus (***water mongoose***)*					X							
*Crocodylus niloticus* (**crocodile**)					X					X		
*Otolemur crassicaudatus* (**greater bushbaby**)					X							
*Hystrix africaeaustralis* (**porcupine**)		3	2				3	4	6		9	1
Leporidae (**hares**)			5	6			4	4		X	47	4
*Struthio camelus* (**ostrich**)					X					X		
*Varanus niloticus* (**nile monitor lizard**)					X							

Data for the table are taken from [66, 71, 78, 79, 81, 82, 86–88] Data on tribes is provided for the bovids. The following site names have been abbreviated in the table: Collingham (Colling.), Good Hope (GH), KwaGandaganda (Kwa.), Mhlwazini (Mhlwaz.), Mzinyashana (Mzinya.), Ndondondondwane (Ndond.).

Nguni farmers began infiltrating the Tugela catchment area in the 5^th^ century AD and by the 10^th^ century were firmly settled in the area [85]. Ndondondwane, KwaGandaganda and Wozi were occupied between the 6^th^ and 9^th^ centuries AD, a period during which hunter-gatherers were still in the area. In marked contrast to the Later Stone Age sites these three Early Iron Age sites were the only ones to produce faunal remains of large, ivory-bearing animals, including hippopotamus and elephant (Table 1; [86, 87, 89]). All three sites were major ivory-working centres, yet only elephant ivory appears to have been used for this purpose [90]. Other large species, such as giraffe and rhinoceros, are absent from these three sites. Domesticates, particularly sheep and goats, made up the bulk of the meat supply, while subsistence hunting was geared towards small game [86, 87, 91, 92]. Maggs [93] has noted that hunting techniques during the Early Iron Age of the highveld grasslands favoured game drives and pitfalls, with the focus being on Alcelaphini herds (e.g., wildebeest, hartebeest). This technique was also used by hunter-gatherers to capture springbuck on the Namaqualand coast during the same time period [94]. A similar pattern is not evident in the Tugela River catchment area (Table 1; also see S1 Table for NISP counts) and it is probable that active hunting with spears and/or bows and arrows rather than game drives was the preferred method of game-meat acquisition. Bone points, most of which are likely the remains of arrowheads [95, 96], are present at all three sites, notwithstanding the general tendency at Iron Age sites for formal bone working to decrease through time in favour of informal utilised pieces [97]. The presence of bone points in the Early Iron Age and their subsequent loss of importance is widely regarded as evidence for contact and changing relations with hunter-gatherers, although, at least at KwaGandaganda, evidence suggests bone points were made on site [87].

## Materials and methods

The development of proteomic techniques has led to a new approach for animal-bone species identification, including one called Zooarchaeology by Mass Spectrometry, or ZooMS [98], using collagen peptide mass fingerprinting [99]. Over the past decade ZooMS has been applied to a wide range of taxa [100], and has been applied to the analysis of bone tools dating back to the Palaeolithic [101].

Eighty-four bone points were selected for inclusion in this study, representing about 16% of the total number of bone points from the eleven archaeological sites (Table 2). All specimens are housed at the KwaZulu-Natal Museum and Amafa Akwazulu-Natali in Pietermaritzburg, KwaZulu-Natal. Specimens were collected, sampled and exported under permit # 13842, granted by Amafa Akwazulu-Natali. As a minimally invasive procedure, we sampled only broken pieces of bone points, and selected shaft fragments based on their overall thickness and ability to withstand the collagen extraction drilling procedure. In selecting thicker shaft fragments, we included ten specimens which showed signs of having been heated. Based on surface observation it was unclear whether the heating damage penetrated deeply or superficially into the bone. Despite numerous successes in non-destructive collagen extraction for ZooMS analysis [12, 13], a previous pilot study from South Africa did not produce results when any of these non-destructive methods were used. Therefore, we decided to extract ~10 mg of bone powder from each specimen. Powder was extracted from the break facet of the bone points under sterile conditions using a 1 mm diameter dental drill at the Wits School of Dentistry.

**Table 2:** Showing the number and percentage of bone point fragments sampled for ZooMS analysis.

Site	Number of cylindrical shaft fragments	Number of specimens sampled	Percentage of sampled specimens
Collingham	86	8	9.3
Driel	14	8	57.1
Good Hope	35	10	28.6
KwaGandaganda	46	11	23.9
Maqonqo	129	12	9.3
Mgede	21	4	19
Mhlwazini	26	2	7.7
Mzinyashana	89	8	8.9
Ndondondwane	12	12	100
Nkupe	51	6	11.8
Wozi	24	3	12.5
**TOTAL**	**533**	**84**	**15.7**

A similar amount of bone powder was also collected from a range of modern reference specimens. Most of our reference specimens were subsampled from the collections housed in the National Museums Scotland (UK). These specimens were either historic specimens, dating from the 19^th^ or early 20^th^ centuries (mostly the unregistered ones), or were recent specimens donated by zoos in the UK. Samples from these specimens were loaned for the ZooMS analyses following National Museums Scotland’s destructive sampling protocols, with no ethical approval being necessary as sampling was of an existing skeletal collection and was minimally invasive. Material for four additional specimens came from a UK-based taxidermist and from the Creswell Crags Heritage Centre (Table 3).

**Table 3:** Complete list of identified peptide markers from the modern comparative material.

Registered Specimen Code	Family	Subfamily	Tribe	Species	Common name	A	B	X1	C	X2	D	X3	F	G	X4
**NMS.Z.2002.212.5**	Bovidae	Antilopinae	Aepycerotini	*Aepyceros melampus*	Impala	1196	1427		1550		2131	2581	2883	3033	3227
**NMS Unregistered**	Bovidae	Antilopinae	Alcelaphini	*Damaliscus lunatus jimela*	Topi	1196	1427		1550		2131	2581	2883	3033	3201
**Unregistered**	Bovidae	Antilopinae	Alcelaphini	*Alcelaphus buselaphus*	Hartebeest	1196	1427		1550		2131	2581	2883	3033	3201
**NMS.Z.1997.22.118**	Bovidae	Antilopinae	Alcelaphini	*Connochaetes taurinus*	Blue wildebeest	1196	1427		1550		2131	2581	2883	3033	3201
**NMS.Z.2002.211.3**	Bovidae	Antelopinae	Reduncini	*Kobus megaceros*	Nile lechwe	1166	1427		1550		2131	2567	2883	3033	3227
**NMS Unregistered**	Bovidae	Antelopinae	Reduncini	*Pelea capreolus*	Grey rhebok	1166	1427		1550		2131	2567	2883	3033	3227
**NMS.Z.2003.138.1**	Bovidae	Antilopinae	Antilopini	*Nanger dama*	Dama gazelle	1196	1427		1550		2131	2581	2883	3033	3227
**NMS.Z.2010.46.43**	Bovidae	Antilopinae	Antilopini	*Eudorcas (Gazella) rufifrons*	Red-fronted gazelle	1196	1427		1550		2131	2581	2883	3033	3227
**NMS.Z.1997.22.62**	Bovidae	Antilopinae	Antilopini	*Litocranius walleri*	Gerenuk	1196	1427		1550		2131	2581	2883	3033	3227
**NMS Unregistered**	Bovidae	Antilopinae	Antilopini	*Antidorcas marsupialis*	Springbok	1196	1427		1550		2131	2553	2883	3033	3227
**NMS.Z.2007.18.2**	Bovidae	Antilopinae	Neotragini	*Neotragus moschatus*	Suni	1196	1427		1580		2131	2581	2883	3033	3227
**NMS Unregistered**	Bovidae	Antilopinae	Neotragini	*Raphicerus campestris*	Steenbok	1196	1427		1550		2131	2581	2883	3033	3227
**Unregistered**	Bovidae	Bovinae	Bovini	*Syncerus caffer*	African buffalo	1208	1455		1580		2131	2581	2853	3075	
**Unregistered**	Bovidae	Bovinae	Tragelaphini	*Taurotragus oryx*	Common eland	1208	1427		1580		2131	2623	2883		
**NMS.Z.2011.165.2**	Bovidae	Caprinae	Caprini	*Ammotragus lervia*	Barbary sheep	1196	1427		1580		2131	2581	2883	3033	
**NMS.Z.2011.147**	Bovidae	Antilopinae	Cephalophini	*Philantomba maxwellii*	Maxwell’s duiker	1208	1427	1514	1580		2131	2581	2883	3059	
**NMS Unregistered**	Bovidae	Antilopinae	Cephalophini	*Sylvicapra grimmia*	Common duiker	1208	1427	1532	1580		2131	2581	2853	3059	
**NMS.Z.2000.378.3**	Bovidae	Antilopinae	Cephalophini	*Cephalophus natalensis*	Natal red duiker	1208	1427	1574	1580		2131	2581	2853	3059	
**NMS.Z.2005.104.2**	Bovidae	Antilopinae	Hippotragini	*Addax nasomaculatus*	Addaz	1196	1427		1580		2131	2581	2883	3059	
**NMS.Z.2001.149.12**	Bovidae	Antilopinae	Hippotragini	*Hippotragus equinus*	Roan antelope	1196	1427		1580		2131	2581	2883	3059	
**NMS.Z.2001.22.2**	Bovidae	Antilopinae	Hippotragini	*Oryx dammah*	Scimitar oryx	1196	1427		1580		2131	2581	2883	3059	
**NMS.Z.2013.61**	Suidae			*Potamochoerus porcus*	Red river hog	1196	1453		1546	1816/32	2131	2579	2883	3033	
**NMS.Z.2012.34.2**	Suidae			*Phacochoerus africanus*	Warthog	1196	1453		1546	1832/48	2131	2579	2883	3033	
**NMS.Z.2000.178**	Canidae	Caninae		*Otocyon megalotis*	Bat-eared fox	1226	1427		1590		2131	2611	2853	2999	
**NMS.Z.2015.121**	Canidae	Caninae		*Lycaon pictus*	African wild dog	1226	1453		1566		2131	2611	2853	2999	
**NMS Unregistered**	Canidae	Caninae		*Canis aureus*	Golden Jackal	1226	1453		1566		2131	2611	2853	2999	
**NMS.Z.2014.96.2**	Felidae	Felinae		*Acinonyx jubatus*	Cheetah	1207	1453		1566		2163	2597	2853	2999	
**NMS.Z.2004.45**	Hyaenidae	Hyaeninae		*Hyaena hyaena*	Striped hyaena	1207	1453		1566		2147	2597	2853	2999	
**NMS.Z.2020.44**	Hyaenidae	Protelinae		*Proteles cristatus*	Aardwolf	1207	1441		1566		2147	2597	2853	2999	
**UoM unregistered**	Bovidae			*Giraffa camelopardalis*	Giraffe	1166	1427		1580		2131		2883	3003	

ZooMS analysis was carried out following the methods described by van der Sluis and colleagues [102]. In brief this involved the decalcification of the aforementioned bone powder with 0.6 M Hydrochloric acid for ~18 h, prior to ultrafiltration into 50 mM ammonium bicarbonate (ABC) using 10 kDa molecular weight cut-off ultrafilter units. This was then digested into peptides using sequencing grade trypsin (Promega, UK) overnight (~18 h) at 37°C and the digests diluted into 0.1% trifluoroacetic acid (TFA) and spotted onto a stainless-steel target plate with an equal volume of matrix solution (10 mg/mL hydroxycinnamic acid in 50% acetonitrile/0.1% TFA) and allowed to air dry following Buckley et al. [103]. Matrix Assisted Laser Desorption Ionization Time of Flight mass spectrometry was performed using a Bruker Ultraflex II instrument over the *m/z* range 700–3,700. Species biomarkers were manually determined for the reference taxa and, together with pre-existing biomarkers in the database [19, 104], used to categorise the archaeological samples upon manual interpretation.

## Results

**Fig 3:**
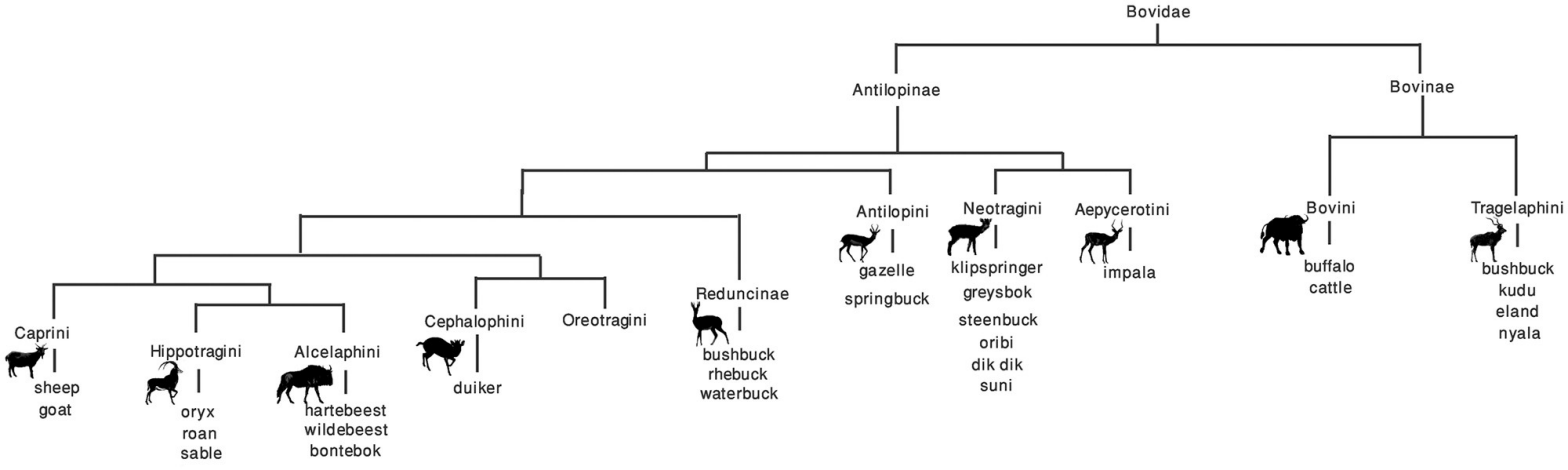
Phylogenetic tree showing southern African representatives of the various tribes and subfamilies of the Bovidae family. Data taken from Groves and Grubb [105].

**Fig 4:**
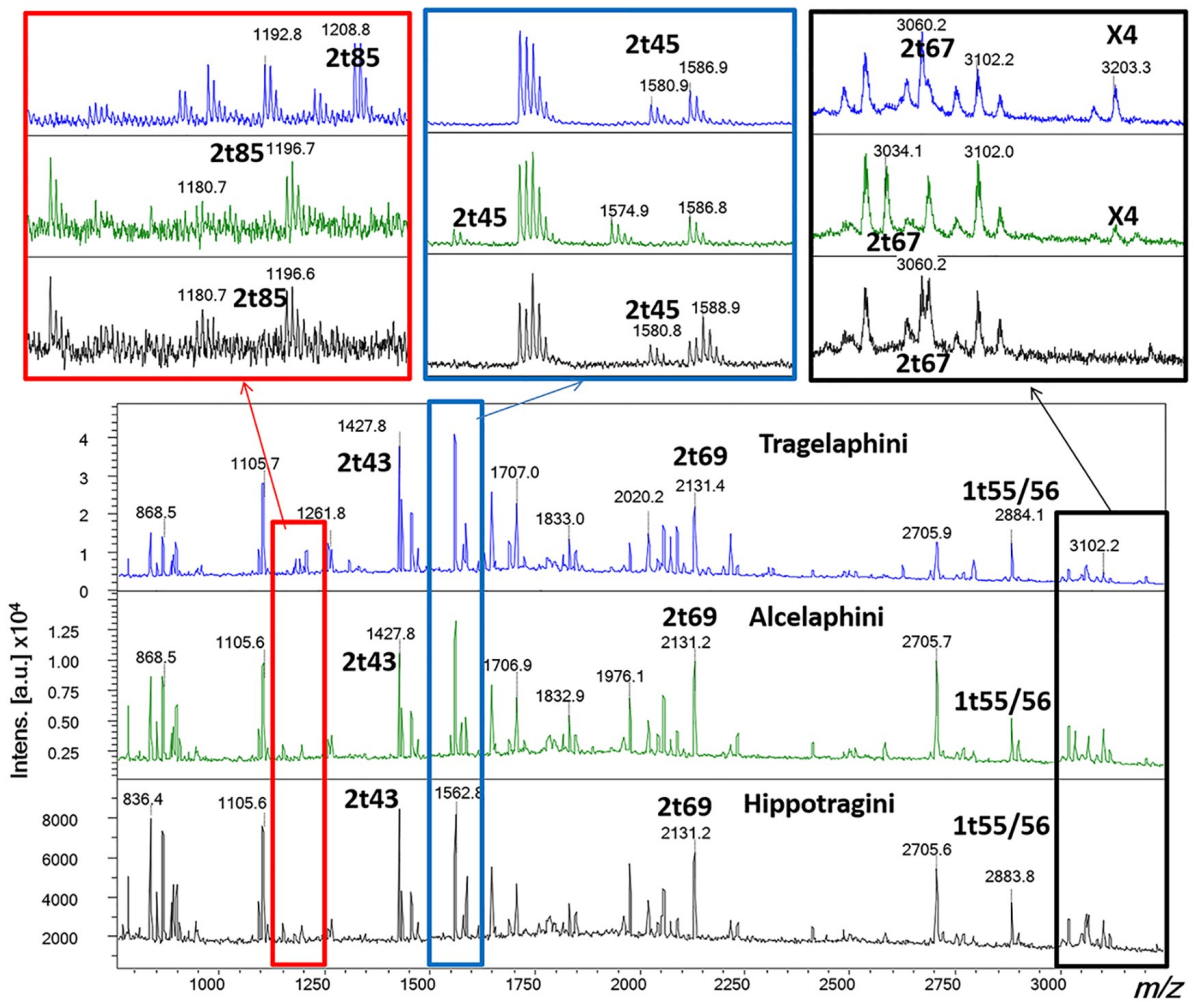
Example of ZooMS collagen peptide mass fingerprint spectra of archaeological Tragelaphini, Alcelaphini and Hippotragini specimens (from top to bottom GH6, MZ5 and MZ2).

Just over half the tested samples returned spectral markers of known provenance (Table 4), allowing us to obtain identifications to the tribe level (Table 5; see S2 Table for details of the peptide markers for the archaeological samples). The relevant southern African species subsumed within these tribes are provided in Fig 3. The most commonly represented group is the Alcelaphini with 18 bone points attributed to this group, followed by Tragelaphini (n = 10), and Reduncini (n = 6) (see Fig 4). In the unmodified fauna sample these three tribes account for only 28% of the total number of bovids present at all sites compared with 42% of the bone points. No species of Aepycerotini, Antilopinior Cephalophini were identified, despite these groups accounting for 65.6% of the total number of bovids identified morphologically in the unmodified fauna across all sites (cf. Table 1). It is apparent that antelopes were the most commonly used mammal for bone-tool manufacture, but one otter, a giraffe, two equids, two hares and three buffalos are also present. All the buffalos come from the Iron Age farmer sites—one from each site.

**Table 4:** Showing the number and percentage of samples from each site that gave positive results.

Site	Number of sampled specimens with positive result	Percentage of sampled specimens with positive result
Collingham	3	37.5
Driel	2	25
Good Hope	4	40
KwaGandaganda	6	54.5
Maqonqo	2	16.7
Mgede	4	100
Mhlwazini	0	0
Mzinyashana	7	87.5
Ndondondwane	10	83.3
Nkupe	2	33.3
Wozi	3	100
**TOTAL**	**43**	**51.2**

**Table 5:** Showing results of the ZooMS identifications (see Table 3 for a complete list of the identified peptide markers, as well as S1 and S2 Figs for spectra representing Reduncini, buffalo, an otter, an equid, a lagomorph and a giraffe).

**Collingham (worked bone n = 169)**					
**Accession #**	**Date**	**Period**	**ZooMS#**	**ID**	**Specimen observations**
N5 BS1	1770–1880 BP	Pre-contact	CHS1	-	
T5 VP1	Undated		CHS2	-	Bone was heated
P5 BSD	1770–1880 BP	Pre-contact	CHS3	Alcelaphini	Bone is coated in poison
S4 BSV2	1770–1880 BP	Pre-contact	CHS4	-	
P4 BSVG	1770–1880 BP	Pre-contact	CHS5	-	
R4 BSV6	1770–1880 BP	Pre-contact	CHS6	*Reduncini*	
S4 FGBS	1770–1880 BP	Pre-contact	CHS7	-	Bone was heated
P5 GAD	1770–1880 BP	Pre-contact	CHS8	Aonyx	
**Driel (worked bone n = 41)**					
**Accession #**	**Date**	**Period**	**ZooMS#**	**ID**	**Specimen observations**
D3 Surf	<1775±40 BP	Pre-contact	DR1	-	
D3 (4)	>1775±40 BP	Pre-contact	DR2	-	
F3 (2)	<1775±40 BP	Pre-contact	DR3	Tragelaphini	
unlabelled			DR4	-	Bone was heated
E3 (3)	1775±40 BP	Pre-contact	DR5	-	
E3 (3) [2]	1775±40 BP	Pre-contact	DR6	-	
E4 Surf	<1775±40 BP	Pre-contact	DR7	Tragelaphini	
E3 (2)	<1775±40 BP	Pre-contact	DR8	-	
**Good Hope (worked bone n = 41)**					
**Accession #**	**Date**	**Period**	**ZooMS#**	**ID**	**Specimen observations**
C4 (2)	<2160±40 BP	Pre-contact	GH1	Leporidae	Bone is polished and discoloured
B4 (5)	<7670±55 BP	Pre-contact	GH2	-	Small mammal limb shaft
C4 (3)	2160±40 BP	Pre-contact	GH3	-	
B4 (4)	>2160±40 BP	Pre-contact	GH4	-	Bone is calcined
B4 (1)	<2160±40 BP	Pre-contact	GH5	-	
B5 (2)	<2160±40 BP	Pre-contact	GH6	Tragelaphini	Base is deliberately squared
B3 (6)	>7670±55 BP	Pre-contact	GH7	-	Small mammal limb shaft
B4 (3)	2160±40 BP	Pre-contact	GH8	-	
C5 SFC (3)	2160±40 BP	Pre-contact	GH9	Leporidae	
C3 (2)	<2160±40 BP	Pre-contact	GH10	Alcelaphini	
**KwaGandaganda (worked bone n = 61)**					
**Accession #**	**Date**	**Period**	**ZooMS#**	**ID**	**Specimen observations**
25c (3)	1395±60 BP	Contact	KWG1	*Syncerus*	Robust, peg-like piece
T2U5 (3)	<1080±60 BP	Contact	KWG2	Alcelaphini	
Sq25 Ext (55–70)	1395±60 BP	Contact	KWG3	Alcelaphini	
F6 (2)			KWG5	Giraffa	
SVP 69	<1080±60 BP	Contact	KWG6	Tragelaphini	
25a Sq3 (2)	1395±60 BP	Contact	KWG7	-	
Sq3 (2)			KWG8	*Equus*	
G2 Pit 1 (55–80)			KWG9	-	Bone was heated
Sq25 E10	1395±60 BP	Contact	KWG10	Tragelaphini	
DB30 (1)	>1395±60 BP	Contact	KWG11		Bone discoloured, possibly from heat
**Maqonqo (worked bone n = 281)**					
**Accession #**	**Date**	**Period**	**ZooMS #**	**ID**	**Specimen observations**
H7 MBS9(G)	>6300 BP	Pre-contact	MQ1	*Equus*	
L7 MBS7	6300 BP	Pre-contact	MQ2	-	Bone was heated
J7 MBS8	<6300 BP	Pre-contact	MQ3	-	
L7 CBS5	7460 BP	Pre-contact	MQ4	-	
L8 DBS3	8670 BP	Pre-contact	MQ5	-	
K6 MBS9	>6300 BP	Pre-contact	MQ6	Reduncini	
L7 MBS3	4960 BP	Pre-contact	MQ7	-	
J7 BS1	3560 BP	Pre-contact	MQ8	-	
L7 MBS2	4140 BP	Pre-contact	MQ9	-	
L7 MBS6	>5680 BP	Pre-contact	MQ10	-	
L7 CBS1	<7460 BP	Pre-contact	MQ11	-	Bone was heated
K7 MBS4	5680 BP	Pre-contact	MQ12	-	Bone has incised decoration
**Mgede (worked bone n = 99)**					
**Accession #**	**Date**	**Period**	**ZooMS#**	**ID**	**Specimen observations**
J4 CBS2	>820±50 BP	Contact	MG1	Tragelaphini	
J3 CBS1	820±50 BP	Contact	MG2	Alcelaphini	
J4 SC1	>820±50 BP	Contact	MG3	Alcelaphini	
J4 CBS1	820±50 BP	Contact	MG4	Reduncini	
**Mhlwazini (worked bone n = 53)**					
**Accession #**	**Date**	**Period**	**ZooMS#**	**ID**	**Specimen observations**
F4 USOBS	2760±50 BP	Pre-contact	MWZ1	-	
D5 BS2	190±45 BP	Contact	MWZ2	-	
**Mzinyashana (worked bone n = 285)**					
**Accession #**	**Date**	**Period**	**ZooMS#**	**ID**	**Specimen observations**
F3 FAP	970±50 BP	Contact	MZ1	-	Circumferential incised decoration
F5 LBS1	2630±60 BP	Pre-contact	MZ2	Hippotragini	Bone is polished
F4 DBS5	1520±20 BP	Contact	MZ3	Tragelaphini	Bone is polished
F4 DBS5 [2]	1520±20 BP	Contact	MZ4	Tragelaphini	
G4 LBS1	2630±60 BP	Pre-contact	MZ5	Alcelaphini	
F4 LBS6	2260±50 BP	Pre-contact	MZ6	Alcelaphini	
F5 LBS6	2260±50 BP	Pre-contact	MZ7	Alcelaphini	Bone is polished
F4 LBS1	2630±60 BP	Pre-contact	MZ8	Tragelaphini	
**Ndondondwane (worked bone n = 12)**					
**Accession #**	**Date**	**Period**	**ZooMS#**	**ID**	**Specimen observations**
K10 SF-10	<1190±50 BP	Contact	NDW1	*Syncerus*	Base is deliberately squared
G12 (1)	1190±50 BP	Contact	NDW2	-	
H10 (2) 3038	1220±50 BP	Contact	NDW3	-	
E10 (1) 2877	1190±50 BP	Contact	NDW4	Alcelaphini	
H10 (1)	1190±50 BP	Contact	NDW5	Alcelaphini	
G11 (2)	1220±50 BP	Contact	NDW6	Reduncini	Base is deliberately squared
H10 (1) [2]	>1190±50 BP	Contact	NDW7	Alcelaphini	
NDO24 P1 west	>1190±50 BP	Contact	NDW8	Alcelaphini	
NDO26 R1 west	>1190±50 BP	Contact	NDW9	Alcelaphini	
NDO midden 1 L1	1190±50 BP	Contact	NDW10	Alcelaphini	
NDO82 1762	<1220±50 BP	Contact	NDW11	Alcelaphini	
NDO I7 (2) 3970	1220±50 BP	Contact	NDW12	Alcelaphini	Base is deliberately squared
**Nkupe (worked bone n = 406)**					
**Accession #**	**Date**	**Period**	**ZooMS#**	**ID**	**Specimen observations**
S11 MBS1	3950±70 BP	Pre-contact	NK1	Tragelaphini	Bone is polished
S10 MBS1	3950±70 BP	Pre-contact	NK2	-	Distal half was heated
R10 WA3B	4590±70 BP	Pre-contact	NK3	-	
R13 LSBS	2480±60 BP	Pre-contact	NK4	Reduncini	
513 VP1	2480±60 BP	Pre-contact	NK5	-	Bone is polished and base squared
513 WA1C	3190±60 BP	Pre-contact	NK6	-	Bone was heated
**Wosi (worked bone n = 24)**					
**Accession #**	**Date**	**Period**	**ZooMS#**	**ID**	**Specimen observations**
G3 T2 OC (2)	1290±50 BP	Contact	WZ1	Alcelaphini	
G4 T1 O6	1290±50 BP	Contact	WZ2	*Syncerus*	
G2 Q4 T4 OC	1290±50 BP	Contact	WZ3	Reduncini	

At KwaGandaganda, Ndondondwane, Nkupe and Wosi we find bone points made from animals that are not present in the unmodified fauna. Three bone points belonging to the Tragelaphini are present at KwaGandaganda and Nkupe, despite no Tragelaphini remains identified in the unmodified bone from these sites. A bone point from KwaGandaganda was identified as giraffe, despite giraffe being absent in the unmodified fauna. Likewise, buffalo bone was used for tool manufacture at Ndondondwane and Wosi, but was not present in the unmodified bone samples. Finally, Alcelaphini were used to make bone points at KwaGandaganda and Ndondondwane (where they comprise the bulk of the bone tools identified), even though not a single animal belonging to this tribe was identified in the unmodified bone samples. This patterning suggests the selective targeting of specific animals for tool manufacture at some sites.

When we view the results from the hunter-gatherer sites, it is apparent that a wider variety of animals are present in the pre-contact levels, although we did get three times as many positive results from these layers than from the contact-period layers (Table 5). Aonyx, leporids, equids and Hippotragini are all represented in the pre-contact levels, but not the contact levels. Alcelaphini and Tragelaphini are the most frequently represented taxa in both contact and pre-contact periods, with the former being far more dominant in the contact period (S3 Table). These are followed by Reduncini in both periods. The only major difference in the representation of taxa is in the low occurrence ones. Nor is there an apparent difference between the Ndaka and Injasuthi social regions (the Toleni social region is represented by only one site: Maqonqo). In both regions Tragelaphini dominate, closely followed by Alcelaphini (S4 Table). When we include the data from the three farmer sites, only equids and buffalos are added to the contact-period species representation. Thus, from the available data it appears that a narrower range of animals was targeted for tool manufacture during the contact period than previously.

As with an earlier ZooMS analysis of bone tools from contact-period sites in Limpopo Province [19], our samples from the three Iron Age sites generally returned better results than those from the Later Stone Age sites (Table 5). The two exceptions are Mgede and Mzinyashana. The reason for this disparity is not fully understood at this stage, as age does not seem to be a factor. Many of the bone points are contemporaneous and some of the oldest bone points, such as those from Maqonqo, yielded positive results. Hoke and colleagues [106] found that the bones from older animals have better isotopic preservation than younger animals, and there are a host of other taphonomic factors that can affect collagen survivability [107, 108]. The proximity of Mgede and Mzinyashana, and Wozi and Ndondondwane to each other may indicate that micro-environment played a role in the preservation of bone collagen at these sites. As might be expected, no bone that had been exposed to heat enough to leave clear signatures on the tools produced a ZooMS result. However, this does further support the utility of screening methods, such as through the use of FTIR [109], which can be done in the field [110], prior to being sent for ZooMS analysis.

## Discussion

The main limitation to a study such as this one is that our inferences must be drawn from a relatively small sample. Owing to size constraints and other practicalities we were unable to sample every bone point from the eleven sites, and those that we did sample did not all return positive results. Nor can we be certain that every species represented at each site was identified in the original archaeozoological studies. A large portion of unmodified fauna from each site was too fragmented to allow for morphological identification beyond the general size category (see Table 1). It is possible that some species are represented in this fraction that were not represented in the morphologically identifiable faunal counts.

There were also important issues with regards the ZooMS analyses themselves, particularly with the analysis of bovid taxa which appear more highly conserved in their collagen peptide mass fingerprints than others, only reaching Tribe level of resolution rather than Genus level, which we have previously obtained even for species of slow population turnover such as elephants [111]. One apparent exception to this is the difficulty in separating the suni (*Neotragus*) from barbary sheep, but we see occasional oddities like this elsewhere with difficulties between *Alces/Cervus/Dama*, etc. Our ZooMS markers build on those previously obtained for African fauna [19], specifically in our ability to now better separate the Alcelaphini (by adding marker: *m/z* 3201) and the Cephalophini (by adding markers *m/z* 1208 and *m/z* 3059) from the other antilopes. We were also able to identify a distinctive peak for Giraffa at *m/z* 3003 (likely reflecting 2t67), and no peak at *m/z* 3033, along with 2t85 (A) at *m/z* 1166.

With the available data, our results appear to show selective targeting of specific animals for tool manufacture at some sites, with a narrowing of the range of selected species during the first millennium AD contact period. This is most marked at the farmer site of Ndondondwane, where most of the sampled bone points were made from Alcelaphini, despite there being no representatives of this tribe being identified in the unmodified fauna [89]. The high incidence generally of bone points made from Alcelaphini is interesting, as, although representatives of this tribe occur at all our sites except Mhlwazini and Nkupe, they never occur in high numbers (Table 1). Nor can the high incidence of Alcelaphini be attributed to game-drive-hunting practices, as other herd animals, such as springbuck and impala, which are also captured by this method [94], are not represented in the bone-point samples. The absence of any Antilopini, Aepycerotini and Cephalophini, which comprise the majority of the unmodified antelope fauna, may be a sampling coincidence, or it may indicate that these animals were deliberately not used to make bone tools. The bone points selected for our sample did not exclude specimens that could have come from animals with a cortical bone thickness in the range recorded for most representatives of these tribes. It is true that Antilopini are generally smaller and more gracile than other taxa, and their cortical bone is therefore thinner. However, the thin bone cortex would not preclude the manufacture of bone points, as some bone needles can be exceptionally thin [80].

The mechanical suitability of bone may also be a determining factor in the choice of which animals to use. The relative proportion of Haversian to plexiform bone plays a role in the ability of bone to withstand compression and torsion [112]. The proportions of these two types of bone differ between groups of animals [113, 114]. The relative proportion of hydroxyapatite to collagen also affects toughness of bone, but this is unlikely to vary significantly amongst bovids [115, 116]. The selection strategies for animal bone at the Middle Stone Age site of Sibudu appear to have favoured artiodactyl bone over perissodactyl bone in the later periods [18], while first millennium AD farmers in Limpopo Province appear to have been cognisant of bone mechanics when selecting raw material for arrowhead manufacture [19]. Such mechanical considerations, however, are not apparent among the eleven samples included in our study. The vast majority of bone points were made from long bones of artiodactyl genera, among which there are no appreciable mechanical differences [115, 116].

In the absence of any obvious mechanical considerations behind the decision of which animals to use for bone-tool manufacture, coupled with the fact that we identified taxa in the bone-point samples from four sites that were not present in the unmodified fauna, one must consider the role of trade and/or socio-symbolic role of animals within societies. Apart from the regional alliance networks mentioned above, there is some evidence of long-distance exchange in the Tugela basin between 7–4 ka [22]. Arrows were a popular exchange item during the nineteenth and twentieth centuries [117] and there is no reason to think this was different in antiquity. However, in no instance did we attribute a bone arrowhead to a taxon outside its natural distribution range. Without more refined identifications to species level, we are unable to properly assess whether people at the different sites may have acquired bone arrowheads through trade networks [118].

The role of animals in structuring elements of human society, on the other hand, is well attested [2, 3], as is the role of bone tools in social networks [15]. The development of selfhood has been shown to be sometimes influenced by our interactions with animals [119], and certain social behaviours of animals may be appropriated by people to express human qualities. Jarman [120] for instance groups southern African antelopes into five classes based on their social behaviours, but does not list all the species he considered. Animals can be incorporated into cosmogenic folk metaphors based on some defining behavioural trait of that animal [42, 60, 121]. For instance, the size and power of elephants and rhinoceroses were used to metaphorically express the power and leadership of African rulers [39]. We did not identify any elephant or rhinoceros bones in our sample of bone arrowheads, but we did find that buffalos were used at the farmer sites. We know that among the Swazi, buffalos are seen as symbols of independence and longevity [5]. Buffalos are also frequently depicted in the hunter-gatherer rock art of Limpopo Province and may have been associated with the mystical rain animal [122]. Their bones are also found in contact-period rain-control sites in the region, supporting this interpretation [36]. Zebras, represented in the bone tools at KwaGandaganda and Maqonqo, are also linked to rain rituals [122]. Buffalos are not alluded to in any of the ethno-historical records of the Limpopo region, reinforcing the idea that different animals may have gained or lost symbolic importance over time due to various factors [32, 69].

Only one carnivoran was identified in the bone point sample (an Aonyx, or otter, from Collingham Shelter). The presence of carnivoran bones in Later Stone Age hunter-gatherer sites is thought to signal ritual activity, more than subsistence [88]. Otters are considered to be messenger animals in Nguni folklore [24], and are one of the few animals, along with buffalos and leopards, thought to be imbued with special, destructive powers [5]. Their skins were often worn by diviners to ward off lightning, while a person who killed an otter was considered to be ‘contaminated’ [5, 49]. The solitary leporid bone tool from Good Hope Shelter is, on the other hand, probably opportunistic, as leporids are not known to have had any special associations and their bones are too small to make ideal arrowheads.

In our results, Alcelaphini are the most frequently identified taxa used to make bone tools (42% of ZooMS identifications), followed by Tragelaphini (23%) and Reduncini (9%). These relative percentages do not differ between the contact and pre-contact periods. During the nineteenth century hartebeests (Alcelaphini) and eland (Tragelaphini) were believed to possess supernatural powers that could aid in various aspects of the physical world, including making it rain [28, 123]. Eland are well known to be quantitatively significant in the rock art of the wider region [30, 124]. Beyond mere archaeological depictions some animals were valued because of certain behavioural traits they exhibited. For instance, wildebeest and hartebeest are known to aggressively charge predators when their calves are threatened [120]. The wildebeest’s swishing of its tail at approaching danger is the reason this body part is used by Nguni diviners in various rituals [5].

Reduncini are the third most frequently represented taxa among the study sample. Although we could not distinguish between *Pelea* and *Redunca*, it is worth noting that waterbuck and reedbuck (the local representatives of Reduncini in the region) have no known special significance among hunter-gatherers, although they were sometimes used to model initiation figurines among the Bantu-speaking farming communities [48]. Grey rhebok (*Pelea capreolus*) on the other hand is the most frequently depicted animal, next to eland, in the rock art of KwaZulu-Natal. In the nineteenth century rhebok potency was thought to be able to influence the movement of game and rain [33, 52, 125]. We know that the rhebok was adopted by the Nguni as an animal totem, and that certain behaviours of the rhebok were incorporated into initiation metaphors [126, 127]. Precisely when this appropriation took place is not known for sure, but it is plausible that it dates to the early period of hunter-gatherer and farmer contact in the first millennium AD. Grey rhebok are highly territorial, maintain harems and are socially stable [128]. It is plausible, though speculative at this stage, that one or more of these attributes found their way into hunter-gatherer and farmer symbolic systems.

Our knowledge of animal symbolism in southern Africa comes primarily from nineteenth and twentieth century ethnography, although it likely has a great antiquity [129]. Animal symbolism from archaeological remains, such as those recovered from rain-making sites in Limpopo Province, are also inferred from references to these ethnographies and recorded folk tales. Animals are routinely represented in art for symbolic purposes and it is common for animal symbolism to find expression in technology.

Here we have offered a glimpse into possible animal symbolism existing in the Tugela basin of KwaZulu-Natal during the pre-contact and contact periods, and the extent to which it translated into the technological sphere in the form of bone-arrowhead manufacture. The collagen spectra from the bone points and reference specimens were, in most cases, too coarse grained to be able to identify to genus or species level, and so we cannot know precisely which animals are represented. But, notwithstanding the level of taxonomic identifications achieved in this study, our results certainly highlight the potential of this line of enquiry and its value for understanding the dynamics of animal symbolism and exploitation in the past.

## Supporting information

S1 Fig ZooMS spectra obtained showing unique markers for buffalo, giraffe and Reduncini. (TIF)

S2 Fig ZooMS spectra obtained showing unique markers for hare, equid and Aonyx. (TIF)

S3 Fig Weighted average percentages of sampled bone tools yielding positive ZooMS results as a factor of total number of bone points at each site. (XLSX)

S1 Table NISP counts for unmodified fauna from nine of the eleven study sites. Note that NISP data are unavailable for Driel and Good Hope shelters.
(DOCX)

S2 Table Peptide markers for the taxonomic identifications of the archaeological material. (DOCX)

S3 Table ZooMS results displayed according to period. (DOCX)

S4 Table ZooMS results displayed according to Tugela basin Later Stone Age social regions. (DOCX)
