# Supplementary figures and images for: Selection preferences for animal species used in bone-tool-manufacturing strategies in KwaZulu-Natal, South Africa
Source: PLoS One. 2021 Apr 1;16(4):e0249296. doi: 10.1371/journal.pone.0249296 (PMC8016335; doi:10.1371/journal.pone.0249296)

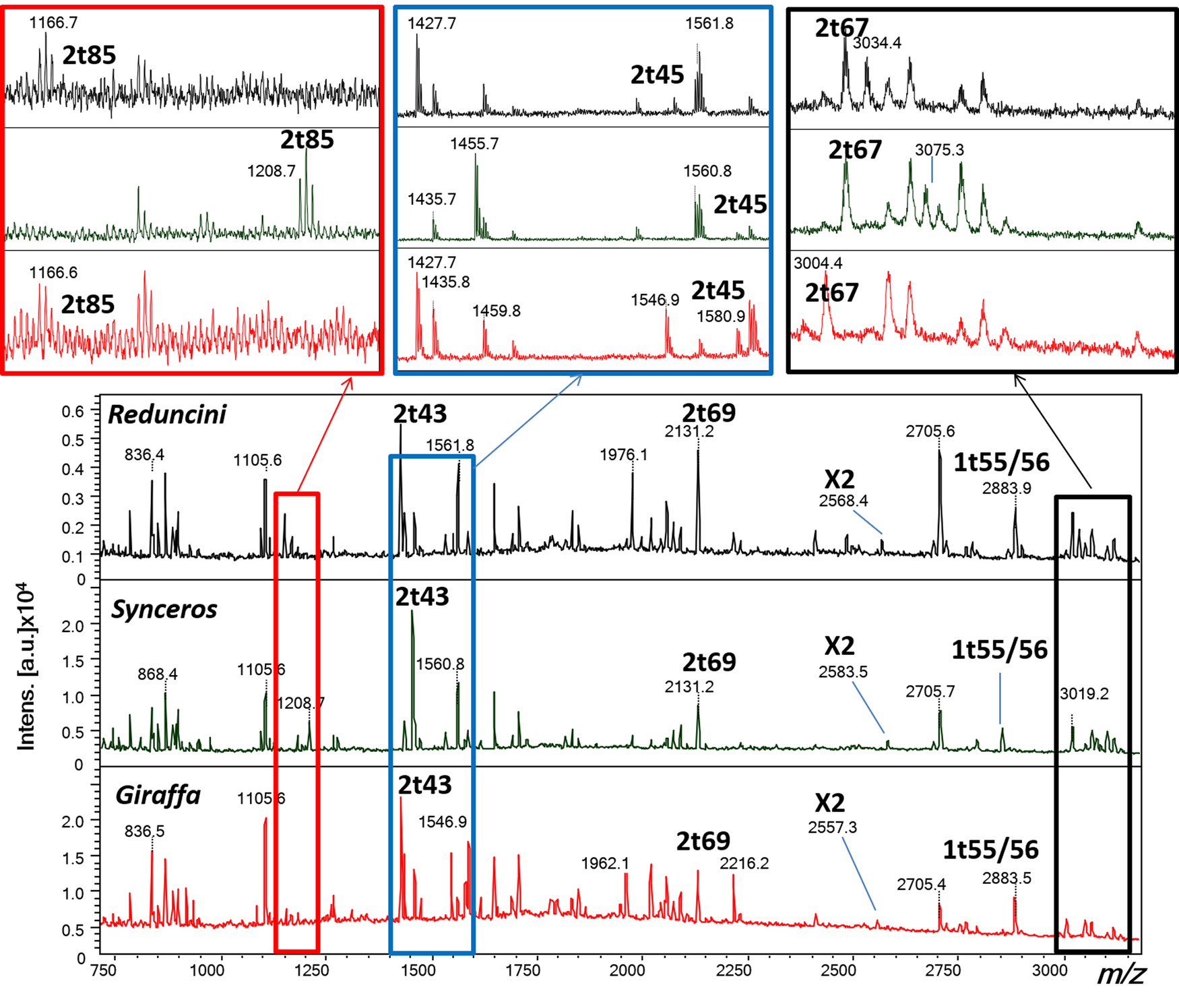

Supplement: S1 Fig — (TIF) [file pone.0249296.s001.tif]

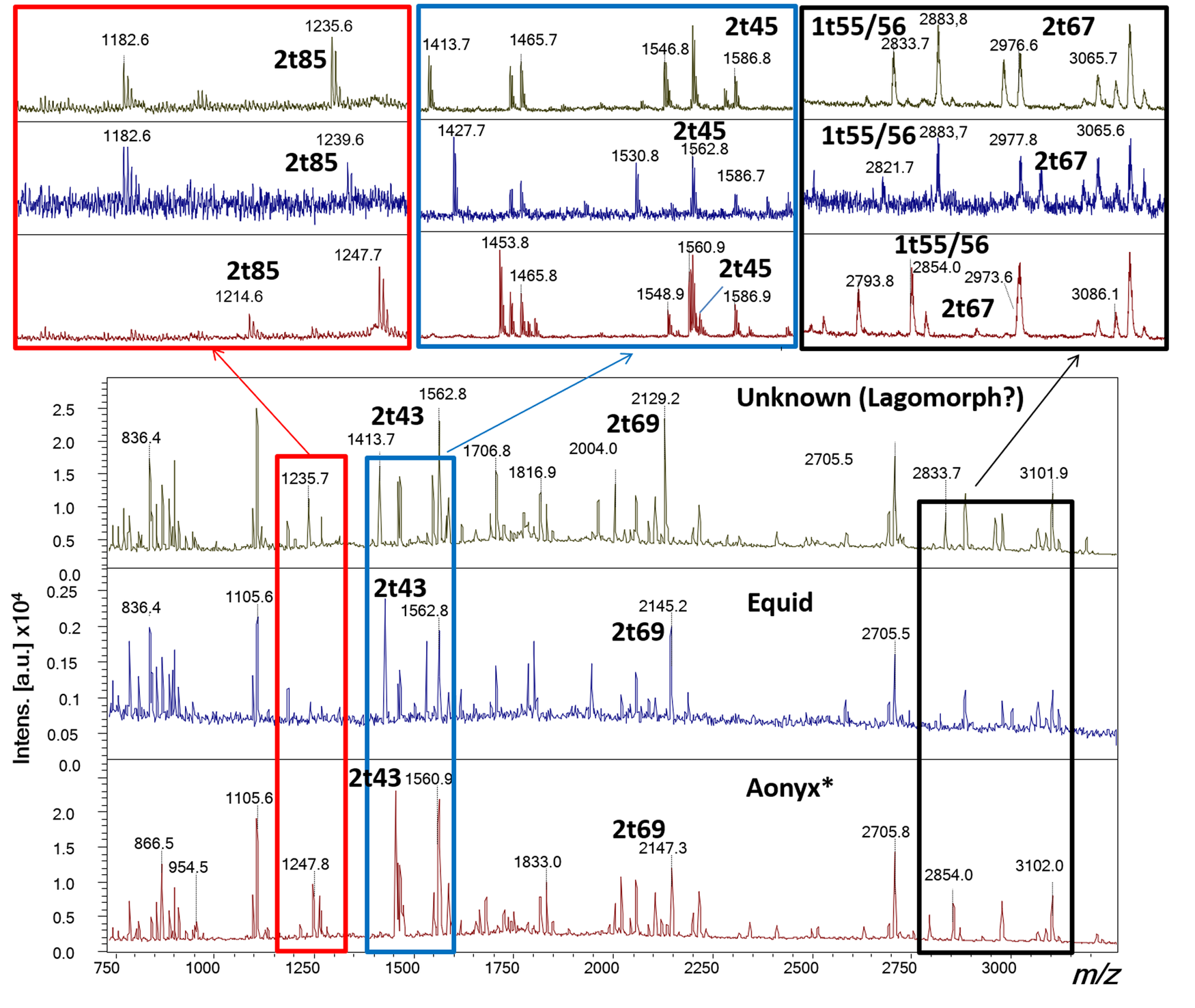

Supplement: S2 Fig — (TIF) [file pone.0249296.s002.tif]
