# Supplementary material for: Selection preferences for animal species used in bone-tool-manufacturing strategies in KwaZulu-Natal, South Africa
Source: PLoS One. 2021 Apr 1;16(4):e0249296. doi: 10.1371/journal.pone.0249296 (PMC8016335; doi:10.1371/journal.pone.0249296)
Supplement: S1 Table — Note that NISP data are unavailable for Driel and Good Hope shelters. (DOCX) [file pone.0249296.s004.docx]

S1 Table. NISP counts for unmodified fauna from nine of the eleven study sites. Note that NISP data are unavailable for Driel and Good Hope shelters.

|  | **Tribe** | **Colling.** | **Driel** | **GH** | **Kwa.** | **Maqon.** | **Mgede** | **Mhlwaz.** | **Mzinya.** | **Ndond.** | **Nkupe** | **Wosi** |
| --- | --- | --- | --- | --- | --- | --- | --- | --- | --- | --- | --- | --- |
| *Homo sapiens* (**human**) |  |  |  |  |  | 1 | 2 |  | 2 |  | 2 |  |
| *Papio ursinus* (**chacma baboon**) |  | 72 |  |  |  | 34 | 3 | 71 | 1 |  | 266 | 7 |
| *Chlorocebus aethiops* (**vervet monkey**) |  |  |  |  | 4 |  |  |  |  |  |  | 9 |
| *Lupulella mesomelas* (**black-backed jackal**) |  | 67 |  |  |  | 35 | 10 | 11 | 14 |  | 59 | 2 |
| *Lycaon pictus* (**wild dog**) |  |  |  |  |  | 2 |  |  | 4 |  | 2 |  |
| *Vulpes chama* (**cape fox**) |  |  |  |  |  | 1 |  |  |  |  |  |  |
| *Canis familiaris* (**dog**) |  |  |  |  | 18 |  |  |  |  | 102 |  | 12 |
| *Crocuta crocuta* (**spotted hyaena**) |  |  |  |  |  |  |  |  | 2 |  |  |  |
| *Parahyaena brunnea* (**brown hyaena**) |  | 1 |  |  |  | 3 |  |  |  |  |  |  |
| *Caracal caracal* (**caracal**) |  | 4 |  |  |  | 5 | 3 | 1 | 10 |  | 81 | 2 |
| *Felis lybica* (**wildcat**) |  | 9 |  |  | 2 | 1 | 21 |  | 4 |  | 91 | 3 |
| *Leptailurus serval* (**serval)** |  |  |  |  |  |  |  |  |  |  |  | 5 |
| *Genetta genetta* (**genet**) |  |  |  |  |  | 2 |  |  |  |  | 14 | 1 |
| *Genetta tigrina* (**Cape genet**) |  |  |  |  | 3 |  |  |  |  |  |  |  |
| *Panthera leo* (**lion**) |  |  |  |  |  | 3 |  |  |  |  | 4 |  |
| *Panthera pardus* (**leopard**) |  | 8 |  |  | 4 | 1 | 1 |  |  |  | 26 |  |
| *Equus quagga* (**zebra**) |  |  |  |  | 2 | 69 | 2 |  | 6 |  | 1 | 1 |
| *Procavia capensis* (**hyrax**) |  | 738 |  |  | 1 | 247 | 47 | 64 |  |  | 578 |  |
| *Proteles cristatus* (**aardwolf**) |  |  |  |  |  | 3 |  |  | 2 |  |  |  |
| *Phacochoerus* sp. (**warthog**) |  |  |  |  | 2 | 304 |  | 1 | 62 |  | 8 | 5 |
| *Potamochoerus larvatus* (**bushpig**) |  | 5 |  |  | 27 | 10 | 16 |  | 59 |  | 50 | 8 |
| *Orycteropus afer* (**aardvark**) |  | 1 |  |  |  | 53 | 4 |  | 49 |  | 12 | 4 |
| *Smutsia temminckii* (**pangolin**) |  |  |  |  | 1 | 5 |  |  | 1 |  |  |  |
| *Mellivora capensis* (**honey badger**) |  |  |  |  |  | 2 | 1 |  |  |  | 2 |  |
| *Giraffa giraffa* (**giraffe**) |  |  |  |  |  | 1 |  |  |  |  |  |  |
| *Loxodonta africana* (**African elephant**) |  |  |  |  | 11 |  |  |  |  |  |  | 291 |
| *Hippopotamus amphibius* (**hippo**) |  |  |  |  | 2 |  |  |  |  | 173 |  | 26 |
| Rhinocerotidae (**white & black rhinoceros**) |  |  |  |  |  |  |  |  | 1 |  |  |  |
| *Ovis/Capra* (**sheep and goats**) |  |  |  |  | 2452 | 34 |  | 1 | 7 | 1964 | 1 | 5399 |
| *Aepyceros melampus* (**impala**) | Aepycerotini |  |  |  | 4 | 48 |  | 5 | 30 |  |  | 3 |
| *Alcelaphus caama* (**hartebeest**) | Alcelaphini | 12 |  |  |  | 24 |  |  | 6 |  |  |  |
| *Connochaetes gnou* (**black wildebeest**) | Alcelaphini | 3 |  |  |  |  | 8 |  |  |  | 7 |  |
| *Connochaetes taurinus* (**blue wildebeest**) | Alcelaphini |  |  |  |  | 45 |  |  | 6 |  |  | 1 |
| *Damaliscus pygargus* (**blesbok/bontebok**) | Alcelaphini | 2 |  |  |  | 2 |  |  |  |  | 14 |  |
| *Antidorcas marsupialis* (**springbuck**) | Antilopini |  |  |  |  |  |  | 1 | 7 |  |  |  |
| *Neotragus moschatus* (**suni**) | Neotragini |  |  |  |  |  |  |  | 11 |  |  |  |
| *Oreotragus oreotragus* (**klipspringer**) | Oreotragini | 22 |  |  |  | 87 | 8 | 158 | 41 |  | 83 |  |
| *Ourebia ourebi* (**oribi**) | Neotragini |  |  |  |  | 69 | 18 | 5 | 64 |  | 108 |  |
| *Raphicerus campestris* (**steenbok**) | Neotragini | 13 |  |  | 4 | 327 |  | 63 | 123 |  | 36 |  |
| *Raphicerus melanotis* (**grysbok**) | Neotragini |  |  |  |  |  | 8 |  |  |  |  |  |
| *Cephalophus natalensis* (**red duiker**) | Cephalophini | 4 |  |  | 4 |  |  | 19 | 10 |  |  |  |
| *Philantomba monticola* (**blue duiker**) | Cephalophini |  |  |  | 44 | 30 |  | 2 | 19 |  |  | 46 |
| *Sylvicapra grimmia* (**common duiker**) | Cephalophini | 2 |  |  | 31 | 259 |  | 16 | 21 | 12 |  | 156 |
| *Hippotragus* sp. (**roan and/or sable**) | Hippotragini |  |  |  |  | 7 | 1 |  | 1 |  | 1 |  |
| *Pelea capreolus* (**grey rhebuck**) | Reduncini | 147 |  |  |  | 38 | 13 | 73 | 25 | 3 | 117 |  |
| *Kobus ellipsiprymnus* (**waterbuck**) | Reduncini |  |  |  |  |  |  |  | 1 |  |  |  |
| *Redunca arundinum* (**reedbuck**) | Reduncini | 5 |  |  |  | 20 |  |  | 46 | 1 |  |  |
| *Redunca fulvorufula* (**mountain reedbuck**) | Reduncini | 8 |  |  |  | 176 | 13 | 2 | 20 |  | 72 | 6 |
| *Taurotragus oryx* (**eland**) | Tragelaphinini | 78 |  |  |  | 36 |  | 3 | 6 |  |  |  |
| *Tragelaphus angasii* (**nyala**) | Tragelaphinini |  |  |  |  |  |  |  |  | 3 |  |  |
| *Tragelaphus sylvaticus* (**bushbuck**) | Tragelaphinini |  |  |  |  | 9 | 16 |  | 2 |  |  | 1 |
| *Tragelaphus strepsiceros* (**kudu**) | Tragelaphinini |  |  |  |  | 10 |  |  | 7 |  |  |  |
| *Bos taurus* (**cattle**) | Bovini |  |  |  | 715 |  |  |  | 8 | 960 |  | 213 |
| *Syncerus caffer* (**buffalo**) | Bovini |  |  |  | 8 | 3 |  |  | 1 |  | 3 |  |
| *BOV I* |  | 169 |  |  | 33 | 786 | 338 | 333 | 316 | 0 | 3417 | 70 |
| *BOV II* |  | 576 |  |  | 37 | 963 | 460 | 330 | 182 | 0 | 2712 | 15 |
| *BOV III* |  | 116 |  |  | 8 | 248 | 57 | 10 | 31 | 0 | 206 | 6 |
| *BOV IV* |  | 0 |  |  | 0 | 7 | 22 | 0 | 4 | 0 | 27 | 0 |
| *Aonyx capensis* (**clawless otter**) |  | 26 |  |  |  |  |  |  |  |  | 10 | 2 |
| *Atilax paludinosus (***water mongoose***)* |  |  |  |  | 1 |  |  |  |  |  |  |  |
| *Crocodylus niloticus* (**crocodile**) |  |  |  |  | 2 |  |  |  |  | 1 |  |  |
| *Otolemur crassicaudatus* (**greater** **bushbaby**) |  |  |  |  | 1 |  |  |  |  |  |  |  |
| *Hystrix africaeaustralis* (**porcupine**) |  | 8 |  |  |  |  | 8 | 9 | 11 |  | 57 | 1 |
| Leporidae (**hares**) |  |  |  |  |  |  | 26 | 18 |  | 2 | 437 | 6 |
| *Struthio camelus* (**ostrich**) |  |  |  |  | 1 |  |  |  |  | 2 |  |  |
| *Varanus niloticus* (**nile monitor lizard**) |  |  |  |  | 7 |  |  |  |  |  |  |  |
