# Supplementary material for: Selection preferences for animal species used in bone-tool-manufacturing strategies in KwaZulu-Natal, South Africa
Source: PLoS One. 2021 Apr 1;16(4):e0249296. doi: 10.1371/journal.pone.0249296 (PMC8016335; doi:10.1371/journal.pone.0249296)
Supplement: S2 Table — (DOCX) [file pone.0249296.s005.docx]

S2 Table. Peptide markers for the taxonomic identifications of the archaeological material.

| **Specimen** | **2t85** | **2t43** | **2t45** | **2t69** | **x2** | **1t55/56** | **2t69** | **Identification** |
| --- | --- | --- | --- | --- | --- | --- | --- | --- |
| CHS1 | X |  |  |  |  |  |  |  |
| CHS2 | X |  |  |  |  |  |  |  |
| CHS3 | 1196 | 1427 | 1550 | 2131 | 2581 | 2883 | 3033 | Alcelaphini |
| CHS4 | X |  |  |  |  |  |  |  |
| CHS5 | X |  |  |  |  |  |  |  |
| CHS6 | 1166 | 1427 | 1550 | 2131 | 2569 | 2883 | 3033 | Reduncini |
| CHS7 |  |  |  |  |  |  |  |  |
| CHS8 | 1247 | 1453 | 1566 | 2147 |  | 2853 | 2973 | Aonyx |
| DR1 | X |  |  |  |  |  |  |  |
| DR2 | X |  |  |  |  |  |  |  |
| DR3 | 1208 | 1427 | 1580 | 2131 | 2623 | 2883 | 3059 | Tragelaphini |
| DR4 | X |  |  |  |  |  |  |  |
| DR5 | X |  |  |  |  |  |  |  |
| DR6 | 1235 | 1427 | 1568 | 2131 |  |  |  | poor |
| DR7 | 1208 | 1427 | 1580 | 2131 | 2623 |  | 3059 | Tragelaphini |
| DR8 | X |  |  |  |  |  |  |  |
| GH1 | 1235 | 1413 | 1580 | 2129 | x | 2883 | 2957 | Lagomorph |
| GH2 | X |  |  |  |  |  |  |  |
| GH3 | X |  |  |  |  |  |  |  |
| GH3 | X |  |  |  |  |  |  |  |
| GH4 | X |  |  |  |  |  |  |  |
| GH5 | X |  |  |  |  |  |  |  |
| GH5 | X |  |  |  |  |  |  |  |
| GH6 | 1208 | 1427 | 1580 | 2131 | x |  | 3059 | Tragelaphini |
| GH8 | X |  |  |  |  |  |  |  |
| GH9 | 1235 | 1413 | 1580 | 2129 |  |  | 2957 | Lagomorph |
| GH10 | 1196 | 1427 | 1550 | 2131 | 2581 |  | 3033 | Alcelaphini |
| KWG1 | 1208 | 1455 | 1580 | 2131 | 2581 | 2853 | 3075 | Synceros |
| KWG2 | 1196 | 1427 | 1550 | 2131 | 2581 | 2883 | 3033 | Alcelaphini |
| KWG3 | 1196 | 1427 | 1550 | 2131 | 2581 | 2883 | 3033 | Alcelaphini |
| KWG5 | 1166 | 1427 | 1580 | 2131 | 2557 | 2883 | 3003 | Giraffa |
| KWG6 | 1208 | 1427 | 1580 | 2131 | 2623 |  | 3059 | Tragelaphini |
| KWG8 | 1198 | 1427 | 1550 | 2145 |  | 2883 | 2999 | Equid |
| KWG9 | X |  |  |  |  |  |  |  |
| KWG10 | 1208 | 1427 | 1580 | 2131 | 2623 | 2883 | 3059 | Tragelaphini |
| KWG11 | X |  |  |  |  |  |  |  |
| MG1 | 1208 | 1427 | 1580 | 2131 | 2623 | 2883 | 3059 | Tragelaphini |
| MG2 | 1196 | 1427 | 1550 | 2131 | 2581 | 2883 | 3033 | Alcelaphini |
| MG3 | 1196 | 1427 | 1550 | 2131 | 2581 | 2883 | 3033 | Alcelaphini |
| MG4 | 1166 | 1427 | 1550 | 2131 | 2569 | 2883 | 3033 | Reduncini |
| MG5 | X |  |  |  |  |  |  |  |
| MG6 | X |  |  |  |  |  |  |  |
| MQ1 | 1198 | 1427 | 1550 | 2131 | x |  | x | Equid |
| MQ2 | 1240? | 1427 | 1580 | 2131 | x | 2883 | x | poor |
| MQ3 | X |  |  |  |  |  |  |  |
| MQ4 | X |  |  |  |  |  |  |  |
| MQ5 | 1166 | 1427 | 1550 | 2131 | 2569 |  | x | Reduncini |
| MQ6 | 1166 | 1427 | 1550 | 2131 | x | x | x | Reduncini |
| MQ7 | X |  |  |  |  |  |  |  |
| MQ8 | X |  |  |  |  |  |  |  |
| MQ9 | X |  |  |  |  |  |  |  |
| MQ10 | X |  |  |  |  |  |  |  |
| MQ11 | X |  |  |  |  |  |  |  |
| MQ12 | X |  |  |  |  |  |  |  |
| MWZ1 | X |  |  |  |  |  |  |  |
| MWZ2 | X |  |  |  |  |  |  |  |
| MZ1 | 1247? | 1427 | X | 2131 | x | 2883 | x | poor |
| MZ2 | 1196 | 1427 | 1580 | 2131 | x | 2883 | 3059 | Hippotragini |
| MZ3 | 1208 | 1427 | 1580 | 2131 | x |  |  | Tragelaphini |
| MZ4 | 1208 | 1427 | 1580 | 2131 | 2623 | 2883 | 3059 | Tragelaphini |
| MZ5 | 1196 | 1427 | 1550 | 2131 | 2581 |  | 3033 | Alcelaphini |
| MZ6 | 1196 | 1427 | 1550 | 2131 | 2581 |  | 3033 | Alcelaphini |
| MZ7 | 1196 | 1427 | 1550 | 2131 | 2581 |  | x | Alcelaphini |
| MZ8 | 1208 | 1427 | 1580 | 2131 | 2623 |  | 3059 | Tragelaphini |
| NDW1 | 1208 | 1455 | 1580 | 2131 | 2581 | 2853 | 3075 | Synceros |
| NDW2 | X |  |  |  |  |  |  |  |
| NDW3 | 1208 |  | X | 2131 | x | 2853 | 3033 | poor |
| NDW4 | 1196 | 1427 | 1550 | 2131 | 2581 | 2883 | 3033 | Alcelaphini |
| NDW5 | 1196 | 1427 | 1550 | 2131 | 2581 | 2883 | 3033 | Alcelaphini |
| NDW6 | 1166 | 1427 | 1550 | 2131 | 2569 | 2883 | 3033 | Reduncini |
| NDW7 | 1196 | 1427 | 1550 | 2131 | 2581 | 2883 | 3033 | Alcelaphini |
| NDW8 | 1196 | 1427 | 1550 | 2131 | 2581 | 2883 | 3033 | Alcelaphini |
| NDW9 | 1196 | 1427 | 1550 | 2131 | 2581 | 2883 | 3033 | Alcelaphini |
| NDW10 | 1196 | 1427 | 1550 | 2131 | 2581 | 2883 | 3033 | Alcelaphini |
| NDW11 | 1196 | 1427 | 1550 | 2131 | 2581 | 2883 | 3033 | Alcelaphini |
| NDW12 | 1196 | 1427 | 1550 | 2131 | 2581 |  | 3033 | Alcelaphini |
| NK1 | 1208 | 1427 | 1580 | 2131 | 2623 | 2883 | 3059 | Tragelaphini |
| NK2 | X |  |  |  |  |  |  |  |
| NK3 | X |  |  |  |  |  |  |  |
| NK4 | 1166 | 1427 | 1550 | 2131 | 2569 |  | 3033 | Reduncini |
| NK5 | X |  |  |  |  |  |  |  |
| NK6 | X |  |  |  |  |  |  |  |
| WZ1 | 1196 | 1427 | 1550 | 2131 | 2581 | 2883 | 3033 | Alcelaphini |
| WZ2 | 1208 | 1455 | 1580 | 2131 | x | x | x | Synceros |
| WZ3 | 1166 | 1427 | 1550 | 2131 | 2569 | 2883 | 3033 | Reduncini |
