# Supplementary material for: Selection preferences for animal species used in bone-tool-manufacturing strategies in KwaZulu-Natal, South Africa
Source: PLoS One. 2021 Apr 1;16(4):e0249296. doi: 10.1371/journal.pone.0249296 (PMC8016335; doi:10.1371/journal.pone.0249296)
Supplement: S3 Table — (DOCX) [file pone.0249296.s006.docx]

S3 Table. ZooMS results displayed according to period

| **Combined pre-contact period site data** | | **Combined contact period site data** | |
| --- | --- | --- | --- |
| **Taxon** | **n** | **Taxon** | **n** |
| Alcelaphini | 5 | Alcelaphini | 13 |
| Tragelaphini | 5 | Tragelaphini | 5 |
| Reduncini | 3 | Reduncini | 3 |
| Leporidae | 2 | *Syncerus* | 3 |
| *Equus* | 1 | *Equus* | 1 |
| Hippotragini | 1 |  |  |
| Hyaenid | 1 |  |  |
