# Supplementary material for: Selection preferences for animal species used in bone-tool-manufacturing strategies in KwaZulu-Natal, South Africa
Source: PLoS One. 2021 Apr 1;16(4):e0249296. doi: 10.1371/journal.pone.0249296 (PMC8016335; doi:10.1371/journal.pone.0249296)
Supplement: S4 Table — (DOCX) [file pone.0249296.s007.docx]

S4 Table. ZooMS results displayed according to Tugela Basin Later Stone Age social regions

| **Ndaka social region** | | **Injasuthi social region** | | **Toleni social region** | |
| --- | --- | --- | --- | --- | --- |
| **Taxon** | **n** | **Taxon** | **n** | **Taxon** | **n** |
| Alcelaphini | 5 | Tragelaphini | 3 | *Equus* | 1 |
| Tragelaphini | 5 | Alcelaphini | 2 | Reduncini | 1 |
| Reduncini | 2 | Leporidae | 2 |  |  |
| Hippotragini | 1 | Reduncini | 1 |  |  |
|  |  | Hyaenid | 1 |  |  |
